# Ipsilateral Rupture of Quadriceps Tendon with Distal Tibia Fracture: A Case Report and Review of the Literature

**DOI:** 10.1155/2017/5012948

**Published:** 2017-05-21

**Authors:** Samik Banerjee, Timothy P. Dooley, James R. Parkinson

**Affiliations:** ^1^Division of Orthopaedic Surgery, Albany Medical Center, Albany, NY 12208, USA; ^2^Division of Orthopaedic Surgery, Stratton VA Medical Center, Albany, NY 12208, USA

## Abstract

Traumatic rupture of the quadriceps tendon by itself is not an uncommon clinical condition. However, its association with concurrent ipsilateral closed distal tibia oblique fracture is exceedingly rare with only one previously reported case in English literature. The dual diagnosis of this atypical combination of injury may be masked by pain and immobilization of the more obvious fracture and may be missed, unless the treating physician maintains a high index of suspicion. Suprapatellar knee pain with or without a palpable gap in the quadriceps tendon and inability to straight leg raise in the setting of a distal tibia fracture should raise concern, but if initial treatment employs a long-leg splint the knee symptoms may be muted. In this report, we describe this unusual combination of injury in a 67-year-old male patient who sustained a trivial twisting injury to the leg. The aim of this report is to raise awareness and emphasize the importance of thorough and repeated clinical examinations in the presence of distracting injuries. Despite the complexity of the problem, standard techniques for quadriceps tendon repair using transpatellar bone tunnels following locked intramedullary rodding of the tibia fracture may lead to optimal outcomes.

## 1. Introduction

Lower extremity tendon lacerations in conjunction with tibia fractures are often seen with high velocity open injuries. However, isolated tendon injures are relatively uncommon with low energy closed tibial fractures. Most studies in literature report associations between tibialis anterior, Achilles tendon, and tibialis posterior tendon lacerations with tibia fractures [1–4]. Isolated traumatic rupture of the quadriceps tendon is an uncommon injury occurring yearly in 1.37/100,000 patients in their sixth and seventh decade and often results from eccentric violent contraction and overloading with forced knee flexion. Some of the common predisposing factors associated with quadriceps tendon ruptures include diabetes mellitus, gout, rheumatologic disease, minor trauma, and attenuation of the tendon with aging.

Although complete quadriceps tendon ruptures can be frequently diagnosed on clinical examination from the triad of acute knee pain, inability to extend the knee, and presence of suprapatellar gap, the diagnosis may be missed due to pain from other distracting injuries such as distal tibia fractures. Only a limited clinical exam is often possible in these situations, and injuries to the quadriceps tendon may be overlooked. Therefore, quadriceps tendon ruptures may potentially be diagnosed intraoperatively, when patients are taken to the operating room after induction of anesthesia for treatment of other injuries, when a more detailed examination is possible. Failure to address a complete laceration of the quadriceps tendon may preclude direct repair and lead to substantial loss of quadriceps strength, patella baja, and considerable disability long-term. Early diagnosis is even more essential when a tibial fracture is considered for nonoperative treatment in a cast, as the diagnosis may be delayed for many weeks. In this report, we describe a patient who sustained an ipsilateral acute complete rupture of the quadriceps tendon with a distal tibia oblique fracture and a supination external rotation ankle injury. The goal of this report is to emphasize the possibility of ipsilateral quadriceps tendon ruptures with oblique distal tibia fractures and to caution orthopaedic surgeons to maintain a high index of suspicion and perform thorough clinical examination to diagnose this injury preoperatively. To the best of our knowledge this association has not been extensively described with one previous report of a similar injury [5]. Additionally, we reviewed the literature on muscle-tendon ruptures associated with tibia fractures.

## 2. Case Report

**Figure 1:**
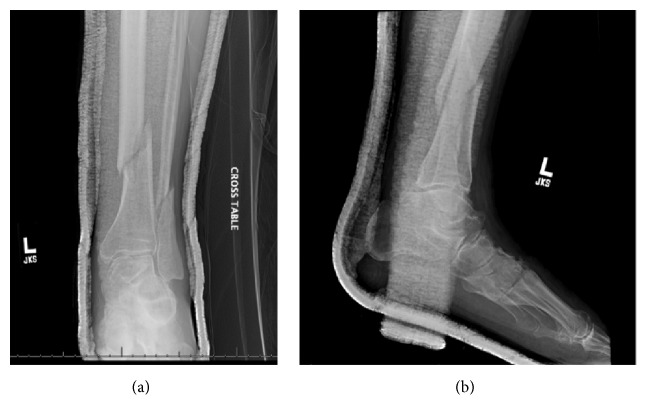
Preoperative AP and lateral radiographic images of the left leg.

A 67-year-old male with past medical history of diabetes mellitus, opioid dependence, and prostate cancer fell down an embankment while attempting to urinate off the side of a road suffering a twisting injury to his left lower extremity. He was seen at an outside emergency department and found to have a closed, comminuted, oblique fracture of left distal tibia-fibula (see Figures 1(a) and 1(b)). The patient was immobilized in a long-leg plaster splint and subsequently transferred to our hospital for definitive care. His left leg was swollen on presentation and tender to palpation over the distal aspect of the leg and globally around the knee. He had weakness of ankle and great toe dorsiflexion on presentation that was baseline from a previous spinal injury. He also had numbness over the left foot, which was present prior to the injury. Distal pulses were found to be intact. A mild to moderate effusion was present in the knee and the patient was unable to complete a straight leg raise, which was felt to be secondary to pain from the tibial fracture and a possible ligamentous knee injury. Initial radiographs revealed an oblique distal tibia and fibula fracture with concern for extension into the tibial plafond. A computed tomography scan was obtained which demonstrated a nondisplaced avulsion of the Chaput's tubercle and a nondisplaced fracture of the posterior malleolus. Due to the substantial comminution of the fracture as well as ipsilateral fibula shaft fracture, operative intervention was recommended.

**Figure 2:**
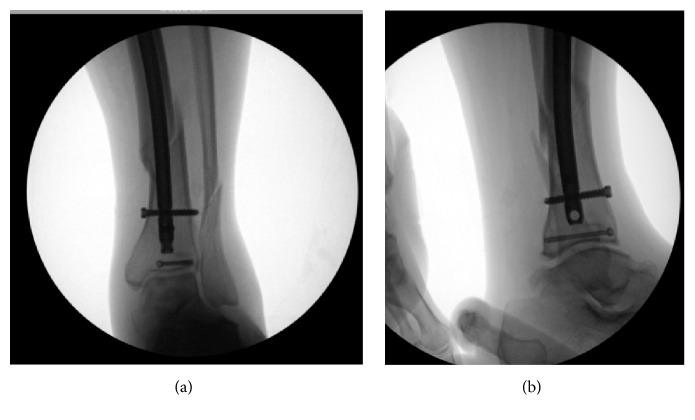
Intraoperative fluoroscopy images following tibial rodding and percutaneous fixation of the posterior malleolus.

**Figure 3:**
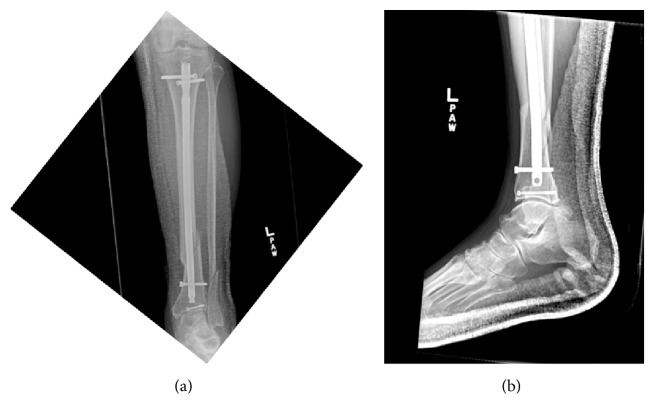
Postoperative radiographs at 4-week follow-up.

**Figure 4:**
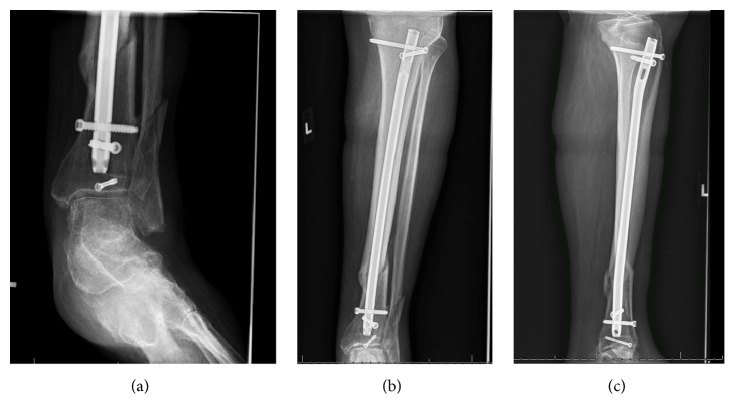
Radiographs at 2-month follow-up showing interval healing with callus formation.

During the surgical procedure, the nondisplaced posterior malleolus fracture was stabilized initially with a percutaneous screw directed anteromedial to posterolateral. The knee was then examined which revealed a substantial gap in the suprapatellar region suggestive of a quadriceps tendon rupture. A midline incision was then made 6 centimeters proximal to the superior pole of the patella to the tibial tubercle. A complete rupture of the quadriceps tendon was found on exploration. The tibia fracture was then stabilized with an intramedullary rod inserted after splitting the patellar tendon in standard fashion (see Figures 2(a) and 2(b)). Following completion of proximal and distal locking ankle external rotation stress test was performed to evaluate for syndesmotic widening. As there was no syndesmotic widening, the fibula fracture was left to heal without fixation. Repair of the quadriceps tendon followed. Krackow stitches were placed in the quadriceps tendon with No-2 Fiberwire® (Arthrex Inc, Naples, Florida) and were passed through 3 drill holes from the superior to inferior pole of the patella. The sutures were then tied with the knee in extension. Postoperatively he was placed in a knee immobilizer and a posterior splint and later transitioned to and ankle equalizer boot and kept nonweight bearing for 6 weeks. He was transitioned to a range-of-motion (ROM) brace allowing knee movement to 90-degree flexion at 4 weeks (see Figures 3(a) and 3(b)). At 8 weeks full movement and partial weight bearing were allowed and the knee brace was discarded (see Figures 4(a)–4(c)). At 3 months, he had regained 0 to 120 degrees of ROM and the fracture had healed completely, and at 6 months following surgery, he had discontinued ambulatory aids and had a lower extremity functional score of 84 points.

## 3. Discussion

Combinations of tendon ruptures with closed isolated tibial shaft fractures are rare injuries that present a diagnostic challenge since physical examination is often limited due patient discomfort, immobilization, and swelling. In addition, suboptimal radiographs in trauma situations are not uncommon and identifying patella baja as a surrogate radiographic indicator of quadriceps tendon injury in lateral knee projections may be exceedingly difficult. Thus, a high index of clinical suspicion is necessary to diagnose quadriceps tendon injuries in patients with tibia fractures. Even with complaints of knee pain and inability to extend the leg, the cause may be mistakenly attributed to musculoskeletal pain from the trauma or an intraarticular ligamentous injury. Primary repair is required once the diagnosis has been made. An advantage of making the quadriceps rupture diagnosed preoperatively is that it may facilitate intramedullary rodding of the tibia fracture using a suprapatellar approach. Access is often easier through the tendon rupture with primary repair performed after the rod has been inserted and locked.

Tibialis anterior, tibialis posterior, and Achilles tendon ruptures are the most commonly reported tendon lacerations seen in conjunction with ipsilateral tibia fractures (see Table 1) [11–15]. It is interesting to note that majority of these tendon ruptures are associated with distal tibia oblique or comminuted fractures thereby suggesting that a twisting mechanism leads to eccentric overload causing failure of tendons concurrently with these fractures. Frequently, these injuries were diagnosed intraoperatively during surgical exploration of the fracture site. In many of these tendon injuries, direct repairs were possible as the lacerated ends were found close to the fracture site. Jarvis and Cannada, in their report of a 48-year-old patient with distal tibia fracture and intraoperatively diagnosed posterior tibial tendon injury, demonstrated excellent outcomes with 5/5 motor strength and AOFAS score 85 points out of 100 points following direct tenodesis of the PTT to the flexor digitorum longus tendon at 1-year follow-up [10].

Concurrent occurrence of a tibia fracture and quadriceps tendon rupture is exceedingly uncommon with only one case reported in literature. Grenier and Guimont described the occurrence of bilateral quadriceps tendon ruptures with isolated left distal tibia fractures in a 39-year-old weightlifter [5]. The authors reported excellent outcomes following direct repair of the quadriceps tendon through drill holes to the patella and limited internal fixation with screws alone and long-leg cast application. The patient regained full range-of-motion (ROM) at 7 months postoperatively. Although we diagnosed the quadriceps tendon rupture intraoperatively, intramedullary fixation of the distal tibia enabled early ROM of the knee in a brace at 4 weeks following surgery. This may have potentially allowed the patient to regain full ROM earlier.

In conclusion, concomitant ipsilateral rupture of quadriceps tendon, closed oblique fracture of the distal tibia, and supination external rotation ankle injuries are rare and to the best of our knowledge have not been extensively reported. Obtaining detailed history of the mechanism of injury and careful clinical evaluation of suprapatellar gap and straight leg raise in patients with oblique fractures of the distal tibia may raise suspicion of this uncommon combination of injuries. Prompt diagnosis may avoid long-term complications and allow early optimal management of this rare combination of injuries. Additionally, when computed tomographic (CT) scans are performed for distal tibia fractures, it may not be unreasonable to obtain sections of the distal femur including knee to supplement a difficult clinical exam for suspected diagnoses of periarticular tendon injuries. Moreover, magnetic resonance imaging of the knee may be obtained preoperatively in the presence of strong suspicion for ligamentous and tendon injuries. Although we did not employ a suprapatellar technique for insertion of tibial intramedullary rod, in the presence of a quadriceps tendon rupture, it may be practical to use this method as it avoids further injury to the extensor mechanism.

## Figures and Tables

**Table 1:** Studies on tendon ruptures after tibial shaft fractures.

Author (year)	Number of patients	Age (years)/sex	Mechanism of injury	Diagnosis	Time of diagnosis	Treatment	Duration of follow-up	Outcomes
Grenier and Guimont [5] (1983)	1	39	Weightlifting	Bilateral quadriceps tendon rupture w/left distal tibia oblique fx	Preoperative	Bilateral repair of quadriceps tendons with limited ORIF with screws for distal tibia fx	5 years	Excellent with FROM in the knee
Mirza and Korber [3] (1984)	1	19 F	MCC	Distal tibial oblique shaft fx w/ipsilateral ATT rupture	1 year following injury	LLC for distal tibia fx; repair of ATT rupture; delayed direct repair after tenolysis	8 months	10-degree active dorsiflexion and normal plantar flexion
Korovessis et al. [6] (1991)	1	26 M		Midshaft tibia w/ipsilateral PTT and FDL rupture	Intraoperative	ORIF tibia and direct repair of PTT and FDL	6 months	10-degree ankle dorsiflexion deficit
Din and Therkilsden [1] (1999)	1	41 M	MCC	Midshaft tibia fx w/ipsilateral ATT rupture	Postoperative	IMR tibia; repair of ATT	NR	NR
Givissis et al. [7] (2004)	1	17	MCC	Distal tibia fx w/ipsilateral ATT rupture	Preoperative	ORIF distal tibia fx w/plates and screws and direct repair of ATT	3 years	Full active dorsiflexion
Mechrefe et al. [8] (2006)	1	32 M	MVC	Distal tibia fx with avulsion fx of tibialis anterior tendon insertion	Intraoperative	ORIF of tibia fx w/plates and screws and repair of ATT avulsion with suture anchors	6 months	Excellent; no bracing needed
Ebrahimi et al. [9] (2010)	1	26 M	MCC	Distal tibia comminuted fx w/ATT rupture	Intraoperative	ORIF distal tibia fx w/plates and screws and direct repair of ATT	12 months	Return to work and recreational activities
Hill et al. [4] (2011)	1	27 M	MCC	Open distal tibia fx w/achilles tendon rupture	Intraoperative	IMR tibia; direct repair of Achilles tendon	5 months	Return to full activity
Jarvis and Cannada [10] (2012)	1	48 M	MCC	Distal tibia-fibula fx (OTA type 43A) w/PTT rupture	Intraoperative	Bridging Ex-fix followed by ORIF tibia and fibula; PTT direct tenodesis to FDL	1 year	AOFAS score 85/100; return to work
Our patient	1	67 M	Fall with twisting injury	Distal tibia oblique fx w/quadriceps tendon rupture w/	Intraoperative	Quadriceps tendon repair and IMR tibia and ORIF posterior malleolus fx	6 months	Return to work and recreational activities

PTT: posterior tibial tendon; ATT: anterior tibial tendon; ORIF: open reduction internal fixation; MVA: motor vehicle accident; MCC: motor cycle crash: IMR: intramedullary rodding; OTA: orthopaedic trauma association; Fx; fracture; NR: not reported; Ex-Fix: external fixator; FDL: flexor digitorum longus; w/: with; Fx: fracture; FROM: full range-of-motion.
